# Comparison of the Benzanthrone Luminophores: They Are Not Equal for Rapid Examination of *Parafasciolopsis fasciolaemorpha* (Trematoda: Digenea)

**DOI:** 10.3390/biom11040598

**Published:** 2021-04-18

**Authors:** Ilze Rubenina, Inese Gavarane, Elena Kirilova, Ligita Mezaraupe, Muza Kirjusina

**Affiliations:** Institute of Life Sciences and Technology, Daugavpils University, Parades Street 1A, LV-5401 Daugavpils, Latvia; inese.gavarane@du.lv (I.G.); jelena.kirilova@du.lv (E.K.); ligita.mezaraupe@du.lv (L.M.); muza.kirjusina@du.lv (M.K.)

**Keywords:** *Parafasciolopsis fasciolaemorpha*, trematode, moose, benzanthrone luminescent dye, confocal laser scanning microscopy

## Abstract

Luminescent derivatives of benzanthrone are becoming more useful based on their light-absorbing and fluorescent-emitting properties. Our previous studies showed that luminescent staining properties of the same benzanthrone dye differ for variable parasite samples. Therefore, two types of benzanthrone dyes were prepared. One has a strongly basic amidine group and a halogen atom, and the other has an amide moiety and a tertiary amine group. Trematoda *Parafasciolopsis fasciolaemorpha* is a liver fluke of a moose (*Alces alces*) and has a significant influence on the health and abundance of the moose population. Staining protocols for parasite *P. fasciolaemorpha* specific organ or organ systems imaging are mostly time-consuming and labor-intensive. The study aimed to compare the fixation technique and the staining protocol by synthesized benzanthrone luminescent dyes to determine detailed morphology, anatomical arrangement of the organ systems and gross organization of the muscle layers of *P. fasciolaemorpha* using confocal laser scanning microscopy. Luminophores were tested for samples fixed in different fixatives. Developed dyes and staining protocol resulting in imaging of all parts of trematode without additional sample preparation procedures, which usually are required for parasite examination. Obtained results confirmed that the most qualitative results could be reached using 3-N-(2-piperidinylacetamido)benzanthrone dye which has amide moiety and a tertiary amine group. Based on obtained results, 3-N-(2-piperidinylacetamido)benzanthrone gave more qualitative parasite visualization than 2-bromo-3-N-(N′,N′-dimethylformamidino)benzanthrone.

## 1. Introduction

Luminescent dyes are becoming popular to label specific biological processes, structures, and molecules [1,2]. Therefore, improvements in dye chemistry are required for the discovery of the helminth’s detailed structure. In a previous investigation by Kirilova et al. [3], various benzanthrone derivatives (with substituted amidine or amine groups in 3-position of benzanthrone core) were applied for visualization of the internal and external structure of freshwater trematodes species such as *Diplostomum spathaceum*, *Diplodiscus subclavatus* and *Prosotocus confusus*. Studied benzanthrone dyes, using AFA fixative, showed good visualization of internal organ systems and body wall of parasites. Additionally, developed luminescent dye 3-N-(2-pyrrolidinoacetamido) benzanthrone was applicable for callus embryo detection [4]. We can explain the obtained results by specific intermolecular interaction between the applied dye molecules and the stained tissues. As it is known that the benzanthrone core, consisting of four fused aromatic rings, has a strong hydrophobicity, primarily interacts with the most lipophilic parts of tissues, namely, with the lipids of cell membranes. At the same time, the dye molecules also contain basic polar groups, which allow them to interact (especially after their protonation with an acidic fixative) with negatively charged groups of tissues, mainly with proteins. Obviously, this combination of interactions of different types contributes to good staining and visualization of the investigated samples. Additionally, benzanthrone luminescent dyes are more photostable, and the development of such slides is required not only for research needs but also for training material preparation. Another important step in a staining protocol creation is sample fixation. The aim of fixation is to keep cellular and extracellular structures as close as possible to the parasite’s structure in vivo and to prevent damages caused by autolysis [5]. The commonly used fixation solutions for further trematodes staining are the ethanol solutions [6]; the Bouin’s solution [7,8,9]; the Carnoy’s solution [10,11,12,13]; the 10% neutral-buffered formalin [14], and the alcohol-formaldehyde-acetic acid (AFA) [15,16]. The choice of applicable staining method and appropriate fixative depends on the study object. The incorrect staining or fixation method has an influence on the interpretation of study results [17]. Continued development of laser technology, digital imaging methods, the availability of brighter and more photostable fluorescent probes and the confocal laser scanning microscopy (CLSM) have made feasible novel experimental approaches for various label fluorescence, multidimensional and live-cell imaging, and microscopy [18]. The improvement of microscopy approaches gives more detailed information about parasite’s organ systems, ultrastructural data for muscle fibers, cell bodies, detailed information about general morphology and gross anatomical arrangement of the organ systems [19,20]. Moreover, CLSM allows re-examining already mounted specimens from helminthological collections [21]. The process of staining is important for the understanding of the parasite’s morphology and species identification. There are numerous staining methods, starting from old and more natural dyes such as carmine and saffron and ending with unnatural dyes, hematoxylin and synthesized aniline dyes [22,23]. Staining protocols using commercially available dyes for CLSM usually are complex and time-consuming, as at least two days are required to obtain results [24]. Through developing specific benzanthrone luminophores for biological object staining, we are suggesting simple and rapid staining protocols that would take up to 20 min.

Moose (*Alces alces*) is a wild definitive host of *Parafasciolopsis fasciolaemorpha* (Ejsmont, 1932) [25]. The hepatic Trematoda parasite is a causative agent of parafasciolopsosis [26,27,28]. The moose liver fluke was first detected in 1932 in Eastern Europe. Nowadays, the parasite was found in roe deer *(Capreolus capreolus*) from Poland [29] and in moose from Belarus [30], from Latvia [31,32], from north-western Russia [33] and from Poland [34]; in red deer (*Cervus elaphus*) from Hungary [35] and from Belarus, and in European bison (*Bison bonasus*) from Belarus [30]. The parasite is leaf-shaped or lanceolate (about 3–7 mm in length and about 1–2.5 mm in width) [36,37]. Moose become infected by ingestion of parasite, which can be swallowed with contaminated water or grass near water basins [34,38,39]. Mature flukes are found in bile ducts, duodenum, and pancreas [28,34,37]. *P. fasciolaemorpha* impact on moose mortality has been clarified in several studies [26,27,28]. Presence of the infection of *P. fasciolaemorpha* in moose has a significant impact on the health of the individual and a potential threat to domestic animals [29]. The present work was aimed to compare the fixation technique and the staining protocol by synthesized benzanthrone luminescent dyes for determination of detailed morphology, anatomical arrangement of the organ systems and gross organization of the muscle layers for *P. fasciolaemorpha* using CLSM.

## 2. Materials and Methods

### 2.1. Synthesis of Fluorophore 2-Bromo-3-N-(N′,N′-Dimethylformamidino)benzanthrone

The molecular formula of 2-bromo-3-N-(N′,N′-dimethylformamidino)benzanthrone (AM323) is C_20_H_15_BrN_2_O. The molecular weight is 379.26 g mol^−1^. The dye AM323 was obtained from 3-amino-2-bromobenzanthrone accordingly to the described procedure [40].

### 2.2. Synthesis of Fluorophore 3-N-(2-Piperidinylacetamido)benzanthrone

The molecular formula of 3-N-(2-piperidinylacetamido)benzanthrone (AZPP) is C_24_H_22_N_2_O_2_. Molecular weight is 370.46 g mol^−1^. The dye AZPP was prepared from 3-(2-chloroacetamido)benzanthrone accordingly to the described method [41].

### 2.3. Fluorescence Measurements

Spectral parameters were measured in eight organic solvents: hexane, benzene, chloroform (CHCl_3_), ethyl acetate (EtOAc), acetone, ethanol (EtOH), N,N-dimethylformamide (DMF), dimethyl sulfoxide (DMSO), and in PBS buffer (pH = 7.4) for solutions with concentrations 10^−5^ M at an ambient temperature in 10 mm quartz cuvettes. All solvents were of p.a. or analytical grade. The absorption spectra were obtained using a UV-visible spectrophotometer Specord^®^ 80 (Analytik Jena AG, Germany). The fluorescence emission spectra were recorded on an FLSP920 (Edinburgh Instruments Co., Ltd., Ediburgh, Scotland) spectrofluorometer using 3-methoxybenzanthrone (QS = 0.56 in acetone [42]) as the reference luminophore [43].

### 2.4. Collection of Adult Parafasciolopsis fasciolaemorpha

Adult Trematoda worms were collected from naturally infected moose (*Alces alces*) livers in the 2018 autumn. Obtained livers were unhealthy; bile ducts were expanded and clogged; cavities were filled with dark yellow liquid. The bile duct and its cavities were cut to collect dark yellow liquid containing parasites. The slimy liquid was rinsed with physiological solution several times until the parasites were completely washed off.

### 2.5. Chemical Fixation

The fixation process is used to prepare parasites for dye binding. Subsequently, the obtained trematodes were fixed in six chemical fixatives. The chemicals, their amounts, pH, fixation and post-fixation times used in this study are shown in Table 1. Fixation was performed at room temperature for all specimens.

### 2.6. Staining Procedure for Parafasciolopsis fasciolaemorpha

Adult Trematoda worms, which were fixed in various chemical fixatives, were used for the staining. The worms were stained with benzanthrone dyes: AM323 and AZPP for 15 min. Then the specimens were washed with 96% ethanol after they were placed in ethanol-xylene (1:1) solution for 8–10 min and cleared by 30 s–3 min with 100% xylene to obtain appropriate transparency controlled under stereomicroscope SMZ800 (Nikon, Japan). Specimens were mounted in the Canada balsam (Sigma-Aldrich) and then were covered with a coverslip (24 × 24 mm), dried and kept in the dark until examination.

### 2.7. Confocal Laser Scanning Microscopy

Finally, the specimens were examined under high-speed multiphoton CLSM Nikon Eclipse Ti-E configured with an A1 R MP microscope system and equipped with a digital sight DS-U3 camera (Nikon, Japan). Slides were observed at various magnifications, from ×40 up to ×600. Autofluorescence was measured with 405 with filter 425–580 nm and 488 nm with filter 500–655 nm wavelengths, and to excite autofluorescence, equal intensities were used. An internal spectral detector performed the registration of the fluorescence signal. The start wavelength for the fluorescence signal registration was chosen to be 20 nm higher than the excitation wavelength until the edge of visible red spectra. Fluorescence was induced by using the following excitation laser wavelengths: (i) λ = 488 nm with the FITC filter (500–550 nm) and (ii) λ = 638 nm with Cy5 filter (662–737 nm). NIS Elements Advanced Research 3.2 64-bit software (Nikon, Japan) was used to process data from CLSM, to make snapshots, Z-stacks (with a 0.9 μm Z step size). The morphological measurements were carried out with a computer program NIS Elements AR Analysis 3.2 64-bit.

## 3. Results

### 3.1. Synthesis

According to the literature and our previous research on the luminescent dyes’ design, benzanthrone derivatives are known as environmentally sensitive fluorophores exhibiting bright from green to red fluorescence depending on their chemical structure both in solutions and in the solid state.

For the development of new efficient luminescent benzanthrone dyes, various organic chemistry methods and synthetic procedures are applied using mainly as initial substances 3-aminobenzanthrone or its derivatives. One of the applied synthesis methods is based on the condensation reaction between the primary amino group of 3-aminobenzanthrone or 3-bromo-9-aminobenzantrone with appropriate unsubstituted and substituted amides in the presence of a dehydrating reagent (phosphorus oxychloride), resulting in new luminescent 3-amidino derivatives [44,45]. The main technique for preparing novel substituted 3-amido dyes is the nucleophilic substitution of the chlorine atom in 3-N-(2-chloroacetamido)benzanthrone by the reaction with an appropriate heterocyclic secondary amine in 1,4-dioxane as solvent resulting in corresponding tertiary heterocyclic amidoamine [41]. In the current research, two novel highly emissive perspective benzanthrone dyes with amidine group (AM323) and with substituted amide group at 3-position of benzanthrone system (AZPP) were selected for visualization purposes.

2-bromo-3-aminobenzanthrone was used as the initial substance for the preparation of fluorophore AM323 by condensation reaction with N,N-dimethylformamide in the presence of phosphorus oxychloride. Obtained red-colored dye is soluble in many polar and non-polar organic solvents.

A second dye, AZPP, was obtained from 3-(2-chloroacetamido)benzanthrone in reaction with piperidine in 1,4-dioxane solution. The prepared yellow-colored dye has better solubility in non-polar organic solvents. Both compounds have excellent emitting properties.

### 3.2. Photophysical Parameters

To fully characterize the prepared luminescent compounds, their optical properties were studied in various media. The UV/vis absorption spectra and fluorescence characteristics (spectra, quantum yields, Stokes shifts) of studied dyes have been recorded in eight organic solvents with a wide range of polarities and in water (PBS buffer, pH = 7.4). The data of absorption and emission band maxima are summarized in Table 2.

In general, it could be seen that absorption spectra do not show substantial variations with solvents: a bathochromic shift in the absorption spectra on changing the solvent from hexane to dimethyl sulfoxide is 35 nm for fluorophore AM323, but for dye AZPP the hypsochromic shift (only 8 nm) is observed. For both studied substances the positions of absorption maxima are situated between 430–465 nm.

The effect of polarity of the solvent on fluorescence is more pronounced than on the absorption spectrum: as the emission spectrum reveals positive solvatochromism when going from non-polar hexane to polar solvent (ethanol, DMF, or DMSO). This is typical spectral behavior for fluorophores with intramolecular charge transfer, which leads to a large dipole moment in the excited state and high emission parameters sensitivity to the polarity of the surrounding [43]. The dye AM323 displays a large bathochromic shift (137 nm) of fluorescence maximum on changing the solvent from hexane (523 nm) to ethanol (660 nm). But dye AZPP demonstrates higher fluorescence bathochromic shift (130 nm), changing the solvent from hexane (531 nm) to dimethylformamide (661 nm). Such a difference between the studied fluorophores can be explained by the specific interaction of the dye molecule with the solvent molecules due to the amino group’s different basicity and the amidino group.

An important photophysical characteristic of fluorescent dye is Stokes shift-difference between positions of the band maxima of the absorption and emission spectra. Stokes shift represents differences in the equilibrium geometries (bond lengths, angles, torsional angles, and vibrational frequencies) of the ground and excited states, i.e., the internal reorganization energy [43].

As seen from Table 2, the Stokes shifts are higher in the case of more polar solvents, indicating distinguished stabilization of the excited state in these solvents.

The maximal Stokes shift value for organic solvents is observed for fluorophore AZPP (up to 222 nm (~7650 cm^−1^) in dimethylformamide solution). However, for AM323 it is 196 nm (~6400 cm^−1^) in ethanol.

### 3.3. Chemical Fixation

In the course of the experiment, 30 trematodes were used for each combination of chemical fixative (in total, six chemical fixatives) and luminophore (in total, two benzanthrone luminophores). Three biological replicates were done during this study. Overall, 1080 trematodes were used for the entire experiment.

It was observed that the specimens’ overall morphology was not changed during the fixation in all cases. None of the fixed samples changed the color after fixation, but the specimen was fixed in Bouin’s solution. As Bouin’s solution contains picric acid, the color of the fixative is yellow, which means that all samples were dyed in yellow. The chemical fixative assessment was performed twice: at the end of the sample preparation process to assess if there are physical changes of trematodes and during microscopy to assess which combination of benzanthrone dye and fixative is the most appropriate (please refer to Table 3).

### 3.4. Staining and Examination of Parafasciolopsis fasciolaemorpha

Staining of parasites was performed in parallel for direct comparison of both dyes. Each fixed trematode was stained 3 times with the same benzanthrone dye.

AZPP dye provided excellent imaging of the whole body of trematode in 40× magnification. The surface, along with spikes, was observed in good quality, oral and ventral suckers were easily detected, the spatial (dimensional) structure was visualized. All three muscle layers-circular, diagonal and longitudinal, were observed at the same time in 100× magnification. In the arrangement of the oral and ventral suckers’ muscle fibers besides circular and longitudinal muscle fibers, also radial muscle layer was visualized. Tegument was visualized in detail like a regular net that covered the parasite. The area where the spike connected to the tegument was visualized. Clear visible parenchyma cells were observed in the tail area. Oral sucker continued in short prepharynx then in the pharynx. The esophagus was split into two caeca. No diverticula were observed, the intestine was smooth. The excretory bladder was clearly visible. The reproductive system was imagined in detail. Radial muscles, cirrus channel, spikes on cirrus surface were observed. The ovary was observed only in few specimens. Uterus was poorly visualized and only with AZZP dye and 70% ethanol combination. Due to fixative eggs were flattened and were observed in the uterus. Vitellaria created follicles located dorsal against the intestine, on both sides of the body, starting from the esophagus to the posterior end. Two irregular testes located under the ventral sucker were observed. Compared to AZPP, the AM323 provided much poorer results. The obtained data are summarized in Table 4. The entire body and structure of the suckers were not clear. Spikes were observed but without visualization of spatial structure. All three muscle fiber types-circular, diagonal, and longitudinal, were observed in 600× magnification, not in 100× magnification. Muscles of suckers as well as tegument were not visualized. Digestive tract: pre-pharynx, pharynx, esophagus, and intestines were obtained in poor quality. From the reproductive system, good visualizations of cirrus, eggs, and testis, vitellaria, ovary and uterus were not detected.

*P. fasciolaemorpha* are relatively thick compared to other trematodes species, e.g., *Prosotocus confusus*, which means that during the fixation process, the *P. fasciolaemorpha* specimen impregnated more Bouin’s or anther fixatives than ticker trematodes. After the fixation in Bouin’s solution, it was observed that fixative could not be washed out completely, and during the confocal microscopy, fixative’s has a major impact on data quality. Based on various benzanthrone dye and chemical fixative combinations experiments, the experiments highlighted that AZPP and 70% ethanol following AZPP and AFA combinations were the most suitable for parasite’s imaging.

The developed method using synthesized AZPP benzanthrone dye is applicable for *P. fasciolaemorpha* examination, make staining protocols less labor-intensive and time-consuming to save resources.

## 4. Discussion

Nowadays, fluorescence bioimaging based on emissive organic molecules has gained great attention as an indispensable tool in research to visualize tissue structures. The spectral changes observed on the binding of fluorophores with cell structures are an important tool for investigating these issues. It becomes necessary to continuously search for new compounds and synthesize new fluorescent dyes covering a wide spectral range of excitation and emission. Therefore, considerable efforts are focused on the development, synthesis, and properties of new luminescent dyes. But the synthesis of new luminescent markers still has several challenges to provide low-toxic dyes for biological objects. In literature, there are described various Trematoda stainings methods such as Gower’s carmine [24], Ehrlich’s acid hematoxylin and Celestin blue-b [13] actin-antibodies and fluorescently labeled phalloidin staining [46,47,48]. Recently we reported that benzanthrone dyes are a useful tool for imaging freshwater trematodes [3], for callus embryo determination [4] and for sex determination of *Trichinella* larva [49]. Therefore, we continue our research on the synthesis of new benzanthrone markers that bind to actin elements to determine internal organs and systems of trematodes.

Derivatives of benzanthrone are well known as p-conjugated compounds with donor–acceptor architectures. Modification of benzanthrone structure has given rise to the synthesis of many derivatives with tenable optical properties. They are typical intramolecular charge transfer luminophores. The optical properties of such molecules depend mainly on the polarizability of the electrons localized in p-bonding molecular orbitals [43]. The polarizability of a molecule is determined mainly by its chemical structure, particularly by the length and the structure of the p-conjugated spacer and the electronic nature of the donors and acceptors attached. As is known, the photophysical properties of 3-substituted benzanthrone derivatives mainly depend on the electron-donating properties of groups connected to nitrogen atoms at the C-3 position [42].

In our previous research, we synthesized several benzanthrone derivatives with various chemical groups such as substituted amidines, secondary and tertiary amines, substituted aminoamides etc. [1,41,44]. These substances luminescence intensely, have high quantum yields, and their spectral properties are sensitive to the local microenvironment’s polarity enabling sensing applications. The spectral properties of the developed fluorescent dyes were investigated in detail [3,4]. As a result, two promising compounds were selected for the current research of the possibilities of studying parasites. In line with our previous studies mentioned before, we prepared AZPP and AM323.

For synthesized derivatives, spectral analysis was undertaken, such as absorption spectra, steady-state fluorescence spectra, Stokes and solvatofluorochromic shifts and emission quantum yields were evaluated and analyzed (Table 2). Developed fluorophores have bright emission in organic solvents from green color in non-polar media to red fluorescence in a polar environment, thus showing excellent fluorosolvatochromism, i.e., sensitivity to the polarity of the medium, that results from solvent relaxation during the excited-state lifetime caused by the essential change of the dye dipole moment after excitation. Parasites need to develop specialized attachment organs for ecto- and endoparasitic survival. Hence a well-developed muscular system is essential. The system provides locomotion, specific feeding, reproduction, and attachment ability within-host [46,47,50,51]. Therefore, synthesized dyes have large Stokes shifts and can be used in super-resolution microscopy of various biological structures. Based on the results obtained, it can be concluded that the developed fluorophores can be used in super-resolution microscopy because super-resolution imaging methods need fluorescent dyes with large Stokes shifts [52]. In continuation of our further work, the developed luminescent dyes were used to visualize *P. fasciolaemorpha* trematode.

**Figure 1:**
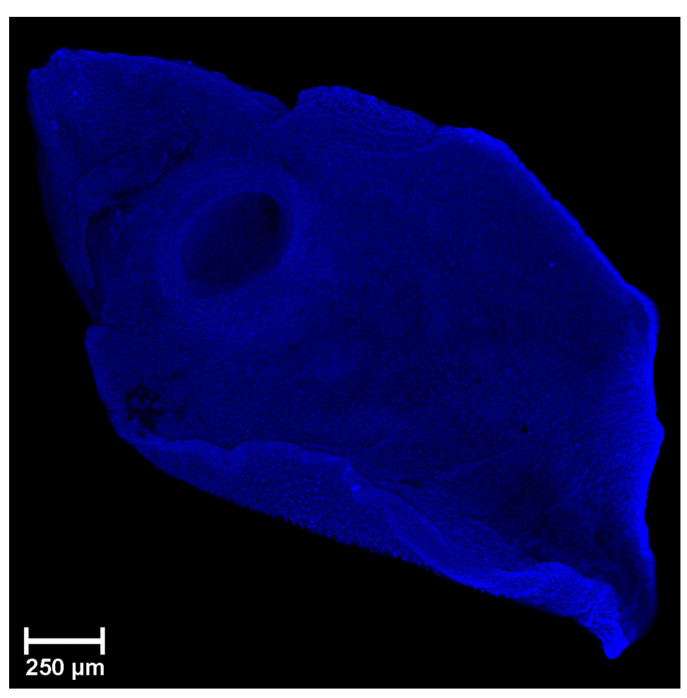
Adult *Parafasciolopsis faciolaemorpha* unstained sample autofluorescence corresponding to different excitation wavelengths (single stack).

Experimental results showed that using laser excitation of 488 nm (with filter 500–655 nm), it was possible to achieve 23-fold attenuation of the autofluorescence signal if we compared it with 405 nm (with filter 425–580 nm) wavelength excitation. To evaluate the autofluorescence, several regions of interest (ROI) were selected, and these were compared to background ROI. Based on obtained data, a 488 nm laser with the FITC filter (500–550 nm) and a 638 nm laser with Cy5 filter (662–737 nm) were the most convenient lasers to suppress unwanted autofluorescence. The autofluorescence image of the sample is shown in Figure 1.

**Figure 2:**
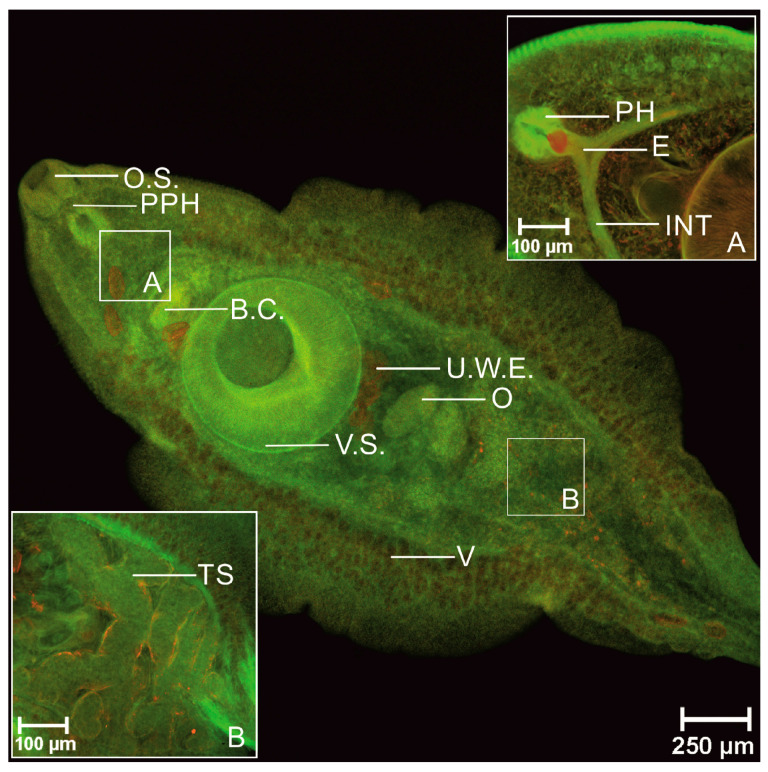
Adult *Parafasciolopsis faciolaemorpha* stained with AZPP dye, fixative AFA (single stack). O.S.—oral sucker, PPH—prepharynx, PH—pharynx, E—esophagus, C—cirrus, V—vitellaria, TS—testes, O—ovary, INT—intestine, V.S.—ventral sucker, U.W.E.—uterus filled with eggs.

**Figure 3:**
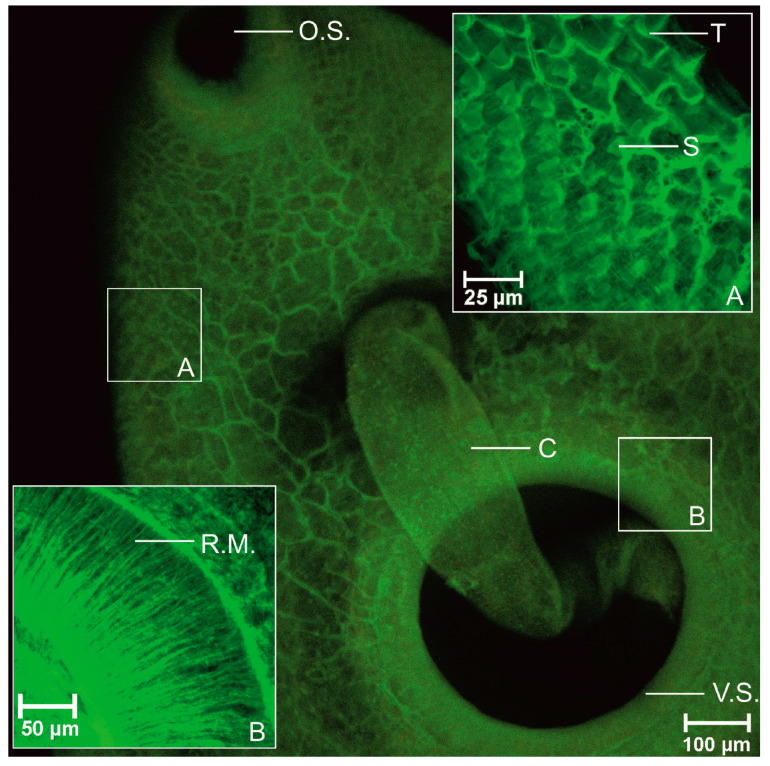
Adult *Parafasciolopsis faciolaemorpha* stained with AZPP dye, ethanol 70% (single stack). O.S.—oral sucker, V.S.—ventral sucker, C—cirrus, T—tegument, S—spikes, R.M.—radial muscle fibers.

**Figure 4:**
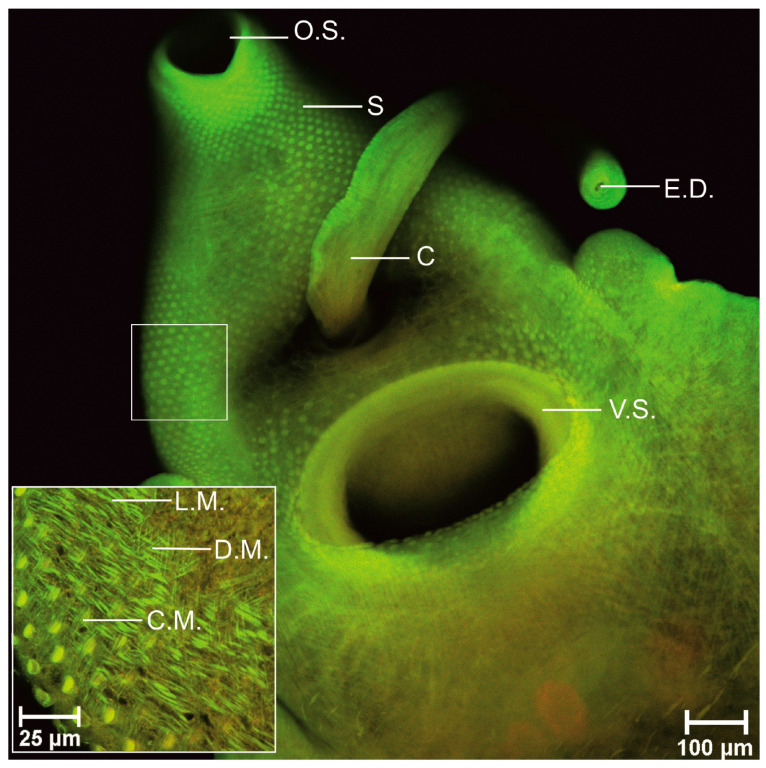
Adult *Parafasciolopsis faciolaemorpha* stained with AZPP dye, fixative 70% ethanol (single stack). O.S.—oral sucker, V.S.—ventral sucker, S—spikes, C—cirrus, E.D.—ejaculatory duct, L.M—longitudinal muscle fibers, D.M.—diagonal muscle fibers, C.M.—circular muscle fibers.

In line with previous studies [19,20,48,53,54,55] our obtained results confirm the arrangement of somatic musculature for adult *P. fasciolaemorpha*. Three main muscle layers were obtained: an outer circular layer, intermediate longitudinal and diagonal layer. Images of Z-series are shown in Figure 2, Figure 3 and Figure 4.

Muscle layers are shown in green. The outer circular layer was organized in flat strips that run parallel to each other; also, the intermediate longitudinal muscle layer was organized in thicker strips than circular muscle fibers. As per below, the circular and longitudinal muscles diagonal muscle fibers organized in bundles were visualized. Diagonal muscles crisscross each other.

We have verified that using our synthesized benzanthrone dyes and developed staining protocol produces similar results to results obtained using fluorescein isothiocyanate or tetramethylrhodamine B isothiocyanate-conjugated phalloidin staining for actin [48]. When comparing our results to those of Kumar et al. [47] study, it shall be pointed out that we detected muscle cell bodies connected to muscle fibers in the diagonal muscle layer. However, we did not observe cell bodies in longitudinal or circular muscles. The musculature of adhesive organs, such as suckers, is mostly very complex, including several muscle types derived from body-wall [20]. Our results showed that both suckers consist of circular, longitudinal and radial muscle layers. A similar conclusion was reached by Terenina et al. [48]. Halton and Maule [50] have demonstrated that the reproductive system and part of the digestive system organs consist mostly of circular muscle fibers, including several longitudinal muscle fibers. After a detailed examination of confocal images, we concluded that we obtained the same results. We also noted that circular muscle fibers are more densely located within cirrus and cirrus sac-like *Fasciola hepatica* [46]. Overall morphology was generally similar reported by Skrjabin [56]. Glycogen reserves were detected in the entire parenchyma below the tegument, however, more in the vitellaria area. Scattered deposits of glycogen were observed in the attachment apparatus. As eggs mostly consist of glycogen and lipids, they have bright fluorescence. Furthermore, glycogen reserves and lipids serve as an energy source, a regulator for cellular activities and building materials for biological membranes [57].

Previous research of parasites was conducted using luminescent dyes of three groups-aminobenzanthrone (P8), amidinobenzanthrones (AM1, AM2, AM4, AM16 and AM323) and aminoamidobenzanthrones (AZP5 and 3-N-(2-piperidinylacetamido) benzanthrone) for various Trematoda species staining. Based on previous study results, only amidinobenzanthrone (AM323) andaminoamidobenzanthrone (AZPP) were used to optimize fixation technique and staining protocol (see Table 5).

Overall view, spikes, oral and ventral suckers of parasites were clearly visualized in all three dye groups. In addition, using protocol with AZPP dye, the dimensional structure of the whole body was clearly visualized. The staining protocol using P8 dye was not suitable for integumentary system visualization. Using dye of amidobenzanthrones group made it to some extent observe the integumentary system, yet images were either not detailed or in pore quality. Compared to dyes mentioned above, usage of aminoamidobenzanthrones group dyes made it possible better visualization of tegument in detail, connected with spikes and all three muscle-fiber types: circular, diagonal and longitudinal. Usage of various dye groups did not affect visualization of Trematoda’s digestive system. Imaging of the reproductive system of adult *P. fasciolaemorpha* was clearer and detailed using AZPP luminophore dye. Opposite amidinobenzanthrones AM1 and AM4 using for *P. confusus* staining show detailed visualization of the reproductive system, and vitellaria was clearly visible.

In contrast to earlier findings, we confirmed that the developed method using synthesized AZPP dye is applicable for *P. fasciolaemorpha* examination, make staining protocols less time-consuming, and save resources.

## 5. Conclusions

Our findings in mutual comparison demonstrate the use of the AZPP luminophore and ethanol 70% or AFA solution as fixatives are a more suitable tool for studies of organic substances–carbohydrates, lipids, and proteins, moreover, for anatomical and muscular arrangement of trematodes than AM323. Our work has led us to the conclusion that specimens fixed in 70% ethanol required an additional 1 to 2 min for sample wash out with xylene compared to specimens fixed in AFA solution. This paper has highlighted that AZPP and 70% ethanol combination is more suitable for external surface and muscle layer examination and AZPP and AFA combination for internal structure assessment. Taken together, the findings suggest that Bouin’s solution is not suitable for *P. fasciolaemorpha* fixation in cases when samples will be used for examination by confocal microscopy.

For further studies, we should investigate the musculature arrangement of the attachment organs of adult *P. fasciolaemorpha*. Our investigations into this area are still ongoing.

## Figures and Tables

**Table 1:** Description of specimen fixation and storage conditions.

					Chemical Fixative	
Chemical Fixative	70% Ethanol	96% Ethanol	AFA Solution	Carnoy’s Solution	Bouin’s Solution	10% Neutral Buffered Formalin
Content of chemical fixative	70% ethanol	96% ethanol	(17:2:1) 85% ethanol: 40% formalin: glacial acetic; pH = 4.5	6:3:1 absolute ethanol: chloroform: glacial acetic acid	(15:4:1) picric acid, saturated aqueous solution: 40% formalin; glacial acetic acid	40% formalin; distilled water; sodium dihydrogen phosphate; sodium hydrogen phosphate
Time of fixation	Until examination	Until examination	2 h	2 h	2 h	Until examination
	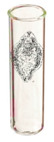	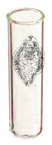	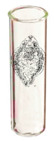	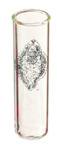	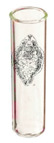	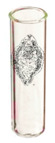
Washing	N/A	N/A	70% ethanol	70% ethanol	70% ethanol	N/A
Chemical fixative	70% ethanol	96% ethanol	70% ethanol	70% ethanol	70% ethanol	10% neutral buffered formalin
Storage	4 °C until required					

N/A not applicable.

**Table 2:** Photophysical parameters of the investigated fluorophores.

Solvent	Dielectric Constant	AM323				AZPP			
		Absorption λ_abs_ (lgε), nm	Emission λ_em_, nm	Φ_0_	Stokes Shift, cm^−1^	Absorption λ_abs_ (lgε), nm	Emission λ_em_, nm	Φ_0_	Stokes Shift, cm^−1^
Hexane	1.89	430 (2.68)	523	0.25	4135	442 (2.62)	531	0.12	3792
Benzene	2.28	446 (2.90)	558	0.23	4500	447 (2.72)	549	0.17	4156
CHCl_3_	4.70	447 (2.94)	593	0.32	5508	445 (2.88)	561	0.58	4647
EtOAc	6.02	448 (2.88)	576	0.23	4960	438 (2.91)	545	0.32	4482
Acetone	20.70	448 (2.87)	603	0.15	5738	438 (2.95)	554	0.57	4781
EtOH	24.30	464 (2.80)	660	0.01	6400	430 (2.97)	562	0.70	5462
DMF	36.70	464 (2.83)	624	0.03	5526	439 (2.92)	661	0.57	7650
DMSO	49.00	465 (3.00)	627	0.02	5556	434 (2.96)	570	0.58	5498
PBS buffer	79.00	469 (2.97)	667	0.01	6329	433 (2.98)	658	0.07	7897

**Table 3:** Comparison of chemical fixatives.

Chemical Fixative	Physical Changes in Specimen	Comments
70% ethanol	−	Optimal concentration of ethanol for trematode sample fixation, no damages to the sample, sample after fixation became a little darker
96% ethanol	+	Specimen became robust; challenging to squeeze between coverslip and slide, sample after fixation became a little darker
AFA solution	−	No physical changes in the specimen were observed
Carnoy’s solution	+	Specimen became impregnated with fixative, which caused enlargement of sample (data not shown)
Bouin’s solution	+	Fixative did not washout; specimen turned yellow
10% neutral buffered formalin	−	No physical changes in specimen observed

− physical changes in specimen was not observed; + physical changes in specimen was observed.

**Table 4:** Comparison of fixatives and benzanthrone dye results obtained by confocal laser scanning microscopy (CLSM).

Characteristic	Benzanthrone Dye	Confocal Microscopy Results Observed (+)/Not Observed (−)					
		Chemical Fixative					
		70% Ethanol	96% Ethanol	AFA Solution	Carnoy’s Solution	Bouin’s Solution	10% Neutral Buffered Formalin
Contours of the whole body are well outlined	AZPP	+	−	+	−	+	+
	AM323	+	−	+	+	−	−
Spikes and layout on the surface are well outlined	AZPP	+	+	+	+	+	−
	AM323	−	−	+	+	−	−
Spatial structure of spikes	AZPP	+	−	+	−	−	−
	AM323	−	−	−	−	−	−
Tegument	AZPP	+	−	−	−	−	+
	AM323	−	−	−	−	−	−
Muscle layers of the body (circular, diagonal, and longitudinal) at the same magnification	AZPP	+	−	+	+	−	−
	AM323	−	−	+	+	−	−
Muscle fibers of oral sucker, radial symmetry	AZPP	+	−	−	−	−	−
	AM323	−	−	−	−	−	−
Muscle fibers of ventral sucker, radial symmetry	AZPP	+	−	−	−	−	−
	AM323	−	−	−	−	−	−
Pharynx, muscle fibers of it can be easily distinguished	AZPP	+	+	+	−	−	−
	AM323	−	−	+	−	−	−
Esophagus can be easily distinguished	AZPP	+	−	+	−	−	−
	AM323	+	−	−	−	−	−
Intestine can be easily distinguished	AZPP	+	−	+	−	−	−
	AM323	−	−	+	−	−	−
Parenchyma cells are well outlined	AZPP	−	−	−	+	−	−
	AM323	−	−	+	+	−	−
Cirrus is well outlined	AZPP	+	−	−	−	−	−
	AM323	−	−	+	−	−	−
Ovary is well outlined	AZPP	+	−	−	−	−	−
	AM323	−	−	−	−	−	−
Uterus filled with eggs	AZPP	+	−	−	−	−	−
	AM323	−	−	−	−	−	−
Vitellaria is well outlined	AZPP	−	−	-	−	−	+
	AM323	−	−	−	−	+	−
Testis can be easily distinguished	AZPP	−	−	+	−	−	−
	AM323	−	−	+	−	−	−
Total +/− (16)	AZPP	13/3	2/14	8/8	2/14	2/14	3/13
Total +/− (16)	AM323	2/14	0/16	7/9	4/12	1/15	0/16

− specified characteristic was not observed; + specified characteristic was observed; +/− ratio of total pluses against total minuses.

**Table 5:** Trematoda staining with dyes from different luminophore groups.

Fluorescent Dye	Chemical Fixator Used	Structure	Object and Description on Stained Systems	References
P8	AFA solution	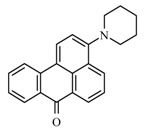	*Prosotocus confusus* adults	[3]
			overall view: spikes, oral and ventral suckers; digestive system: prepharynx, pharynx, esophagus, intestine; reproductive system: uterus with eggs, cirrus	
AM1	AFA solution	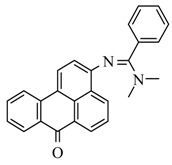	*Prosotocus confusus* adults	[3]
					overall view: spikes, oral and ventral suckers; integumentary system: radial and longitudinal muscle fibers; digestive system: prepharynx, pharynx, esophagus, intestine, excretory bladder, excretory pore; reproductive system: ovary, testis, vitellaria, uterus with eggs, cirrus
AM2	AFA solution	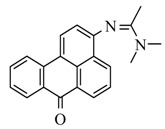	*Diplostomum* sp.	[3]
			overall view: oral and ventral suckers, pseudosuckers, holdfast, calcareous bodies; digestive system: pharynx, esophagus, intestine; primary excretory system (very bright)	
AM4	AFA solution	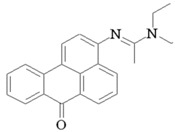	*Prosotocus confusus* adults	[3]
			overall view: spikes, oral and ventral suckers; integumentary system: diagonal and longitudinal muscle fibers in poor quality; digestive system: prepharynx, pharynx, esophagus, intestine; reproductive system: ovary, testis, vitellaria, cirrus, eggs	
AM16	AFA solution	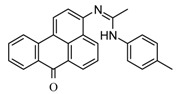	*Diplostomum* sp.	[3]
			overall view: oral and ventral suckers, pseudosuckers (very bright), holdfast; digestive system: pharynx, esophagus, intestine; primary excretory system (very bright)	
AM323	AFA solution	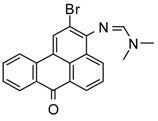	*Parafasciolopsis fasciolaemorpha* adults	Current study
			overall view: spikes, oral and ventral suckers; integumentary system: three muscle layers and radial muscles of suckers in poor quality; digestive system: prepharynx, pharynx, esophagus, intestine, reproductive system: testis, eggs, cirrus	
AZP5	AFA solution	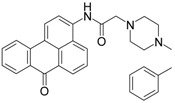	*Parafasciolopsis fasciolaemorpha* adults	[55]
			overall view: spikes, oral and ventral suckers; integumentary system: tegument not in details, three muscle layers and radial muscles of suckers; digestive system: prepharynx, pharynx, esophagus, intestine, reproductive system: testis, eggs, cirrus	
AZPP	AFA solution	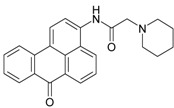	*Parafasciolopsis fasciolaemorpha*	Current study
			overall view: spikes, oral and ventral suckers in detail, the dimensional structure of the whole body; integumentary system: tegument (in details) connected with spikes, three muscle layers and radial muscles of suckers in details and very bright; digestive system: prepharynx, pharynx, esophagus, intestine, reproductive system: ovary, testis, vitellaria, uterus with eggs, cirrus and its canal	

## Data Availability

The data presented in this study are available in this article.
